# Oral contraceptives and colorectal cancer risk - A meta-analysis and systematic review

**DOI:** 10.1016/j.amsu.2022.104254

**Published:** 2022-10-03

**Authors:** Fadi Abusal, Mohannad Aladwan, Yazan Alomari, Saja Obeidat, Salah Abuwardeh, Haya AlDahdouh, Qotada Al-shami, Qusai Odat

**Affiliations:** a General Surgery, Al Bashir Hospital, Amman, 11151, Jordan; b Obstetrics and Gynaecology, Al Bashir Hospital, Amman, 11151, Jordan; c General Surgery, Royal Medical Services, Amman, 11855, Jordan; d Obstetrics and Gynaecology, Alyarmouk Hospital, Irbid, 21243, Jordan; e 5th Year Medical Student, University of Jordan Hospital, Amman, 11942, Jordan; f General Surgery, Princess Basma Teaching Hospital, Irbid, 21110, Jordan; g Obstetrics and Gynaecology, Princess Basma Teaching Hospital, Irbid, 21110, Jordan

## Abstract

There is limited understanding of the potential relationship between the risk of colorectal cancer and oral contraceptive use among women of different ages. Further investigation on the issue helps develop an informed choice of contraception. Data for this meta-analysis were derived from case-control and cohort studies of colorectal cancer and oral contraceptive use conducted between June 2000 and May 2022. The studies had a very high heterogeneity, as shown by an I^2^ of 99%, and a confidence interval of 95% was considered significant. Other results from the meta-analysis were as follows; Heterogeneity: Chi^2^ = 585.13, df = 6 (P < 0.00001). A test of the overall effect of ever use versus never use of oral contraceptives was Z = 21.85 (P < 0.00001). All the studies had a pooled risk ratio (RR) of 0.53. The use of oral contraceptives is associated with reduced risk of developing colorectal cancer. There is a need for further research into the biological mechanisms underlying these relationships, which may lead to insights into potential preventive interventions for colorectal carcinogenesis in women. The keywords used to locate studies included in this meta-analysis include Keywords targeting oral contraceptives included oral contraceptive pills, and birth control pills. Search keywords targeting colorectal carcinogenesis included neoplasms, tumors, or colon and rectal cancer.

## Highlights

• Oral contraceptive use in woman does affect the risk of developing colorectal cancer.
• Colorectal cancer has many identifiable and nonidentifiable risk factors.
• OCP usage in women of various age groups shown a lower risk of colorectal cancer development than those who never used OCPs.
• Regarding the population at risk for colorectal cancer due to a variety of reasons, many studies have deemed OCP usage safe.

## 1 Introduction

The third most common cancer diagnosed every year is colorectal cancer with more than one million new cases being diagnosed globally [1]. The incidence of this type of cancer has lower incidence in females compared to males of similar age groups. This observation is attributed to higher estrogen levels in females which confers protection to females [2]. Epidemiologic studies have come up with findings consistent with this hypothesis reporting a 20–40% lower incidence of cancer of the colorectum among ever-users compared to never-users of oral contraceptives [[3], [4], [5], [6]]. Several scientific studies have suggested the role of hormonal and reproductive factors on colorectal carcinogenesis in women. This association was first suggested after observing high numbers of colorectal cancers in nuns from different denominations in the US [7]; and an inverse relationship between parity and colorectal cancer.

The study was conducted to identify the association between marital status and human carcinogenesis among 31,658 catholic nuns from 41 denominations in the United States (US). Cancer mortality rates in nuns of old age were considerably higher than in controls. The incidence of colorectal cancer and other types of cancers among nuns in the post-menopausal ages showed increased frequency. Together with other findings, this observation implicated the effect of infertility and marital status. It also suggested that cancers of the large colon, rectum, and specific reproductive sites had common biological mechanisms with hormonal characteristics. Other studies have been conducted on the subject since this observation was made five decades ago. Regarding this topic of ever-use versus never-use of oral contraceptives, a meta-analysis done by Ref. [8], up to June 2000 showed that the cumulative relative risk (RR) of developing cancer of the colorectum was O.82 (95% confidence interval, CI, 0.74–0.92) from all studies combined - 0.81 from eight case-control studies and 0.84 from four case-control studies. From the meta-analysis, colon carcinogenesis (OR = 0.63; 95% CI = 0.45–0.87) and rectal carcinogenesis (OR = 0.66; 95% CI = 0.43–1.01) were inversely proportional to ever-use of oral contraception. The duration of oral contraceptive methods application had an inverse relationship to the risk of developing colonic but not rectal cancer. However, no relationship between duration/period of ever use of oral contraceptives and lowered risk of colorectal carcinogenesis was observed in 6 cohort studies [[9], [10], [11], [12], [13], [14]], and 14 from case-control investigations [2,8,8,15,16,[16], [16], [17], [18], [19], [20], [21], [22], [23], [24]]. Most of these studies have found an inverse relationship between oral contraception methods and increased risk of colorectal cancers. Colorectal cancer mortality has declined more in women than in men in some developed countries over the last two decades.

Birth control pills/oral contraceptives are medications that contain hormones that are taken orally to avert pregnancy. The administration of these drugs avert pregnancy by preventing ovulation and prevent viable sperm from reaching the cervix. Some common oral contraceptives in developed countries like the United States (US) contain synthetic versions of the female hormones progesterone and estrogen - often referred to as a combined oral contraceptive. The other type of oral contraceptive contains only progestins/synthetic progesterone and is, therefore, referred to as the mini pill.

Most research on the association between cancer risk and oral contraceptives comes from population-based case-control studies and large prospective cohort studies. However, observational studies cannot be used to establish that exposure to oral contraceptives increases or reduces the incidence of colorectal cancer among women of different age groups. This may be the case because women who actively take birth control pills may differ from those who do not take oral contraceptives in other ways apart from their birth control pill use. It is, therefore, possible that these other disparities are responsible or can explain these differences in cancer risk. In general, however, these studies have been a source of consistent evidence supporting the hypothesis that oral contraceptives are associated with a reduced risk of contracting a range of cancers like ovarian, endometrial, and colorectal cancers.

On the other hand, they have shown that women who take oral contraceptives face an increased risk of developing breast and cervical cancers. Oral contraceptives influence cancer risk through different mechanisms. Endogenous and exogenous female sex hormones, i.e., estrogen and progesterone, may lower the risk of colorectal cancers in multiple ways, including reducing the number and frequency of ovulation females experience throughout their lifetime, therefore, reducing the time of exposure to potentially carcinogenic sex hormones as in the case of ovarian cancer. Second, oral contraceptives may suppress endometrial cell proliferation as in endometrial cancer. Finally, birth control pills reduce bile acids in the blood, as seen in women under regular oral conjugated estrogens regimens [25]. This study evaluated menstrual and reproductive factors after chronic exposure to exogenous and endogenous hormones in several prospective studies targeting colorectal cancer.

The incidence of colorectal cancer among women was investigated in relation to reproductive and menstrual factors. This cohort study featured 93,676 women, among which 1149 colorectal cancer cases were reported over 11.9 years. In this study, having had two or more children was inversely correlated with the risk of developing colorectal cancer. Females previously exposed to oral contraceptives had a lower risk of colorectal cancer (HR = 0.74, 95% CI: 0.63–0.86); there was however, no association observed between the duration of oral contraceptive use and the risk of colorectal carcinogenesis (4 years vs. one year: HR = 0.94, 95% CI: 0.67–1.32). The study results concluded that prior use of oral contraceptives and parity were the two significant factors that could be directly linked to a lower risk of developing colorectal cancer among women of different age groups. Several studies have sought to investigate this phenomenon and have provided information on the association between colorectal cancers and the use of combined oral contraceptives (OCs).

Endogenous and exogenous hormones could also influence the risk of developing colorectal cancer, as indicated by several epidemiological, metabolic, and animal data studies. A recent Swiss case-control study [26] on 373 hospitals and 131 women with colorectal cancer reported an OR of 0.8 for ever-use oral contraceptives. This occurred without the presence of a consistent relationship with the duration of use of oral contraceptives. A Wisconsin, USA, case-control study [27], that included 1122 colon cancer cases, 366 rectal cancer cases, and 4297 controls, had an overall odds ratio for ever-use of 0.89. There was no difference between rectal (OR = 0.87) and colon cancer (OR = 0.87). A Canadian case-control study on 1404 cases of colorectal cancer and 1203 controls revealed a reduction of risk in carcinogenesis associated with ever-use of oral contraceptives OC (OR = 0.77) with little to no evidence of an association between duration of OC use and carcinogenesis [28].

A cohort study by the Oxford Family Planning Association on 46 cases of colorectal cancer and 17,032 women also reported similar results [29]. On the other hand, the Oxford Family Planning association cohort study [29] on 46 reported colorectal cancer cases, including 17,032 women, found no association between the cancers with oral contraceptive use. In a Shanghai cohort study on 267,400 women in Shanghai, China, that reported 455 women having colon cancer, it was found that the relative risk (RR) for women who had used oral contraceptives before was 1.09. There was the absence of any relationship between OC use and an increased risk of carcinogenesis [30]. In a follow-up on an oral contraceptive cohort study conducted by the Royal College of General Practitioners' (35 years follow-up on 46,000 women), 323 cases of colorectal cancer were reported, and this corresponded to a relative risk of 0.72 for women who had used oral contraceptives previously [31]. Also, a nested case-control study was conducted within the cohort and found 146 colorectal cancer cases [31].

This study found a greater reduction of risk for current oral contraceptive users (OR = 0.38) than former users (OR = 0.89). Lin et al. [32], found little evidence supporting a duration-related reduction of risk for ever-use of OCs (RR = 0.67) in an 11-year cohort study that included 267 cases of colorectal cancer and 39,680 women [32]. conducted a study including 89,835 Canadian women in a breast cancer screening program, where they found 1142 colorectal cancer cases, with a relative risk of 0.83 for ever-use of oral contraceptives. However, they could draw no relationship between reduced cancer risk and oral contraceptive use. This issue is, therefore, still open for research. The IARC Monograph found little evidence supporting OC's lack of carcinogenic effect on colorectal cancers [33].

To quantify the association between colorectal cancer and oral contraceptive use, it was necessary to conduct this meta-analysis and systematic review, which includes select published articles up to June 2000. Eight articles were handpicked for conducting this meta-analysis. Of these, 3 were case control studies [[26], [27], [28]] while four were cohort studies [3,29,30,32].

## 2 Tools and methods

### 2.1 Protocol

The protocol of this meta-analysis was prepared in lieu of the investigation according to the standards of systematic reviews outlined by the Preferred Reporting Items for Systematic Reviews and Meta-Analyses (PRISMA). The criteria of inclusions followed these guidelines without interference from external factors. Therefore, this systematic review and meta-analysis results have been generated credibly to offer genuine and accurate insights into the topic question. The systematic review and meta-analysis use the PRISMA extension published in the Cochrane Handbook for systematic reviews and interventions – Chapter 4 [34].

### 2.2 Search strategy

Databases of Cochrane Library, PubMed, and EMBASE were used to identify publications assessing the relationship between oral contraceptive use and the incidence of colorectal cancers among women through an electronic search. This meta-analysis was conducted in May 2022, and studies focusing on the oncological outcomes in women of different ages who used oral contraceptives (birth control pills) were considered. Articles considered for this study included published research papers on colorectal cancer in English language up to June 2016. To maximize the search strategy results, this meta-analysis incorporated three techniques to create three search queries to be used on Cochrane Library, PubMed, and EMBASE. These techniques included keywords, Boolean operators, truncations, and field tags. The search strategy adhered to the PRISMA statement and publication bias standards outlined by Ref. [35]. The search strategy keywords were derived from the basic concepts of oral contraceptives, and colorectal carcinogenesis. Keywords targeting oral contraceptives included oral contraceptive pills, and birth control pills. Search keywords targeting colorectal carcinogenesis included neoplasms, tumors, or colon and rectum cancer. The search used title/abstract [tiab] as the designated field tags for the two search queries. It further incorporated Boolean operators AND and OR and truncations to complete the search queries.

### 2.3 Search string

The studies used for this meta-analysis were retrieved from the stated databases using the following search string: [‘colorectal’ OR ‘colon’ OR ‘rectal’ OR ‘rectum’] AND [‘oral contraceptives’ OR ‘exogenous hormones’] AND [‘cancer’ OR ‘neoplasm’] AND [‘case –control study’ OR ‘cohort study’].

### 2.4 Eligibility criteria

In this meta-analysis, case-control studies and cohort studies were included for analysis. The inclusion criteria focused on studies that evaluated the oncological outcomes of individuals/patients with a history of use of oral contraceptives. Studies were eligible only if information had been obtained directly from each woman, and oral contraceptives could be distinguished from other hormone replacement treatments [15], [16], [17], [18], [2], [8], (19), [16], [20], [21], [22], [23], [8], [24]. No studies used for this meta-analysis were assigned quality scores, there was no a priori exclusion of studies based on weakness of design or data quality. The exclusion strategy used for this analysis involved performing sensitivity analyses which revealed studies that only provided crude estimates of the outcomes of interest. Also, when multiple studies were published for the similar populations, the most recent/informative study was included for this meta-analysis.

### 2.5 Data extraction

Before extracting the data, the included studies were assessed for the risk of bias according to the methodological standards outlined in the Cochrane Handbook of Systematic Reviews of Interventions [34]. A standardized excel sheet was prepared and refined purposely to extract data that would be relevant for this systematic review and meta-analysis. The same investigators involved in selecting studies were also involved in retrieving relevant information for this review. All pertinent outcomes were extracted, and all the units were standardized for data pooling and comparability. Some of the information extracted included the type of study, number of subjects (cases and controls), the incidence of colorectal cancers (test and control groups), the prevalence of OC use among women and the confounding factors allowed for in the study, as shown by Table 1, Table 2. The primary analysis concerned comparing ever versus never-use of oral contraceptives.

**Table 1:** Data extracted from case control studies.

Reference	Country; Study acronym	Cases + Incidence of OC use	Controls + Incidence of OC use	Age (median)	Confounding
(26)	Switzerland	131 (14)	373 (63)	62	Age, education, family history of CRC, parity, fiber intake, and physical activity
(27)	Wisconsin, USA	1488 (426)	4297 (1968)	47	Family history of colorectal cancer, physical activity, and hormone replacement therapy
(28)	Canada	1404 (705)	1203 (680)	48	Physical activity, body mass index (BMI)

**Table 2:** Data extracted from Cohort studies.

Reference	Country; Study acronym	Cohort Size	Follow-up years	Age	CRC Cases	Confounding
[29]	Oxford FPA, UK	17,032	30	25–39	46	Physical activity, alcohol intake, Body mass index
[30]	China	267,400	10	36–70	655	age, parity
[32]	WHIS, USA	39,680	11	52–60	267	Body mass index, physical activity, smoking status, red meat intake, alcohol consumption,
[36]	Canada	89,835	16	40–59	1142	Physical activity, dietary variables, body mass index (BMI)
[35]		93,676	11.9	50–79	1149	Age, family history of colorectal cancer, race/ethnicity, education level, hormone therapy status, a history of diabetes, smoking status

*FPA - Family Planning Association; WHIS - Women's Health Initiative Study.

### 2.6 Data analysis

Data analysis was performed on Review Manager version 5.4 (RevMan 5.4) to find the association between the use of oral contraceptives (birth control pills) and the incidence of colorectal cancer among women. Two study populations (test and control) were analyzed to demonstrate the relationship between the use of oral contraceptives and the incidence of colorectal cancers. The number of cancer cases in each section of two populations was the main outcome of interest. A confidence interval of 95% was used for data analysis. Details extracted from each study include, study design, number of subjects (cases and controls or person-years), prevalence of oral contraceptive use, and confounding factors. Primary analysis of the data involved making comparisons between ever-users and never-users of oral contraceptives. Also, wherever possible, the influence of recency and duration of use was assessed. In most studies, the primary outcome was the combination of cancers of the rectum and colon, but some outlined colon cancer only, while some studies considered colon and rectum carcinogenesis as two separate outcomes. No study was assigned a quality score. Also, no studies there was no exclusion of studies a priori for weaknesses of data quality or study design.

The most significant measure of effect for most studies was the relative risk (RR) for cohort studies, which was approximated by the odds ratio (OR) in case-control studies, with a 95% confidence interval (CI). Summary estimates of the RR were derived using fixed effects models. Heterogeneity of the studies was evaluated using a I^2^ test [37]. In this study, funnel plots are useful in determining Publication bias [38]. The RRs, ORs, and CIs were abstracted from published papers by giving preference to estimates adjusting for multiple contradictory factors. within the case wherever variable relative risks weren't obtainable, they were obtained by computing them exposure distribution as highlighted within the articles. A weight adequate to every study's exactness was used to calculate the average weight of the calculable relative risks. For the 2 styles of studies used in the meta-analysis, summary estimates were calculated individually, as well as in combination. A forest plot was given within which each study was plotted using a square. The square's center projection on the underlying scale corresponded to the calculable relative risk (RR). The realm of the plotted square was directly proportional to the inverse of the variance of the log of the relative risk [39].

## 3 Results

### 3.1 Study selection

**Fig. 1:**
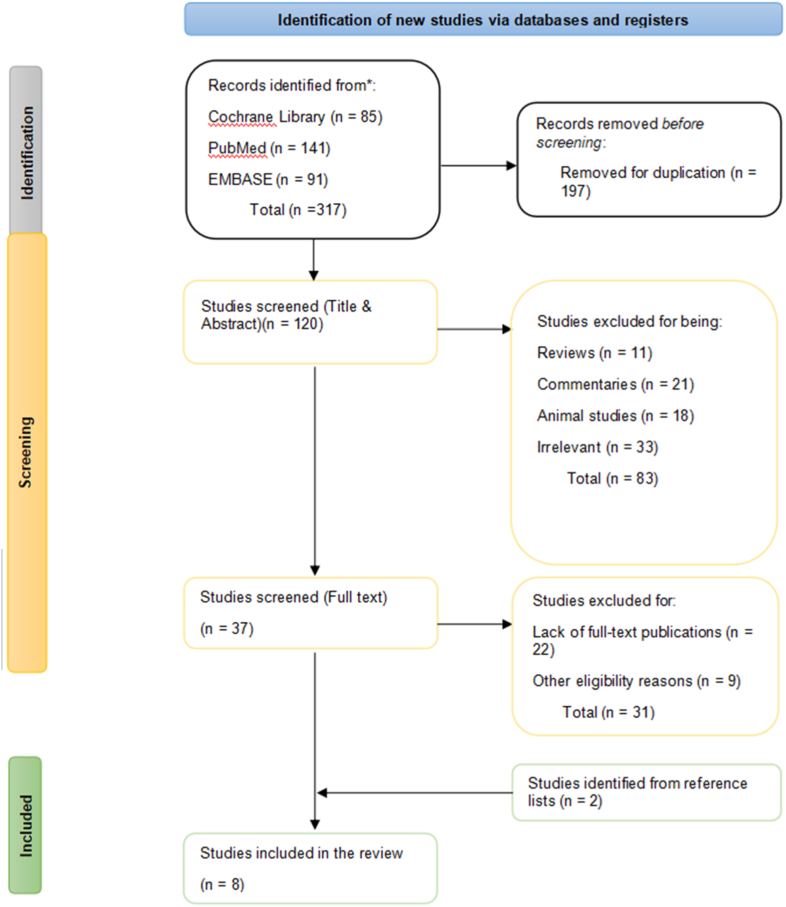
PRISMA flow diagram.

A total of 317 studies were screened and considered for inclusion. A total of 197 studies were excluded due to duplication, and 120 studies were submitted for a title, and abstract screening. 11 systematic reviews and meta-analysis, 21 commentaries, and 18 animal studies were excluded. Also, 33 studies were excluded for irrelevance in the intervention used, not measuring the targeted outcomes and use of undesired population. The next stage was a full-text screening of 37 studies, eliminating 22 for lack of full-text publications. Further eligibility reasons such as the method of reporting used, lack of a control group, and the outcomes reported in the results sections were used as grounds for the elimination of a further 31 studies. A total of 6 studies were then left for inclusion, with an additional two studies identified in reference lists of former systematic reviews and meta-analyses being brought in to make 8. Fig. 1 below shows a PRISMA 2020 flow diagram for updated systematic reviews, summarizing the selection criteria outlined above.

### 3.2 Statistical results

**Fig. 2:**
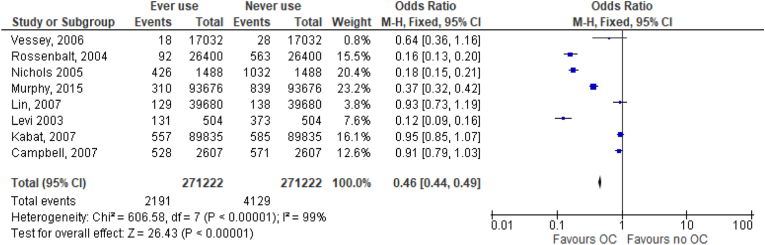
Forest plot of the odds ratio of ever versus never oral contraceptive use and colorectal cancer incidence.

**Fig. 3:**
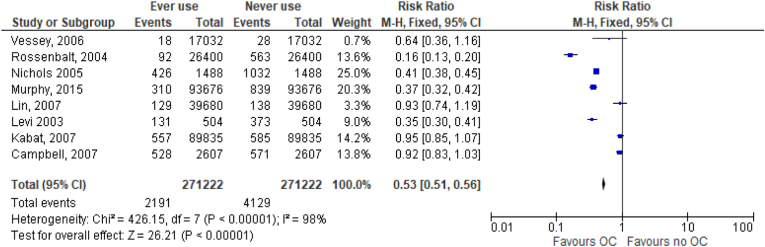
Forest plot of the risk ratio of ever versus never oral contraceptive use and colorectal cancer incidence.

Eight studies (4 good, 3 fair, 1 poor quality) evaluated the association between use of oral contraceptives among women and the incidence of colorectal cancer. Of these, 3 were case–control studies [26,28,40] while 5 were cohort studies [25,29,30,32,36]. Fig. 2 shows the results showing a decrease in the risk of colorectal cancers among women who ever used oral contraceptives compared with women who never used oral contraceptives. Data extracted from these studies was analyzed and represented on forest plots as shown on Fig. 2, and on a funnel, plot as shown on Fig. 3. Fig. 2 shows the odds ratios (ORs), and the incidence of colorectal cancer cases against the total number of study participants. Two study populations were considered to demonstrate the relationship between the use of oral contraceptives and the incidence of colorectal cancers. The number of cancer cases in each section of two populations was the outcome of interest. The studies had a very high heterogeneity as shown by an I^2^ of 99% and a confidence interval of 95% was considered significant. Other results from the meta-analysis were as follows; Heterogeneity: Chi^2^ = 585.13, df = 6 (P < 0.00001). A test of the overall effect of ever use versus never use of oral contraceptives was Z = 21.85 (P < 0.00001). Fig. 3 shows a forest plot illustrating the pooled risk ratio of 0.53.

## 4 Discussion

The strength of proof for the impact of oral contraceptive pill use on large intestine cancer incidence was moderate. Results were mostly consistent across studies, and the summary estimate showed high preciseness with a good CI. Future studies won't probably have an impression on the direction of the impact however could slightly influence the magnitude of the effect. The strength of proof for the duration of therapy was insufficient; the check was underpowered and that we found vital heterogeneity. From this meta-analysis and systematic review, there was a 21% reduction within the risk of developing large intestine cancer among ever-users of oral contraceptives. 2191 cases of colorectal cancer among 271,222 study participants reported using oral contraceptives. The use of oral contraceptives is associated with 0.46 RR of developing colorectal cancer. There was pronounced reduction in RR in subjects that showed recent oral contraceptive pill use, however, this impact wasn't duration dependent. This finding is consistent with other meta-analyses [[3], [4], [5], [6],^8^] which accepted studies on the same topic up to June 2000. From this meta-analysis, no relationship was established between period of ever use of oral contraceptives and lowered risk of colorectal carcinogenesis from 6 cohort studies [[9], [10], [11], [12], [13], [14]] and 14 from case-control investigations [2,8,8,15,16,[16], [16], [17], [18], [19], [20], [21], [22], [23], [24]]. Most of these studies have found an inverse relationship between the use of oral contraception methods and increased risk of colorectal cancers.

**Fig. 4:**
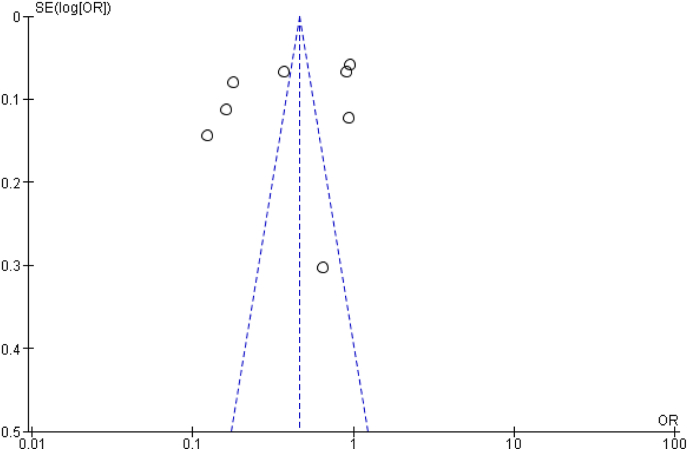
Funnel plot showing the relationship between oral contraception and incidence of colorectal cancers.

There was a very high heterogeneity of the studies probably because case-control and cohort studies were considered for use in the meta-analysis to create a common odds ratio and risk ratio pools. On publication bias, it was decided not to search for unpublished data, and to exclude papers that did use personal questionnaires to gather data. Studies with small sample sizes or null results were not included as they are very likely to be published [41]. There was no significant asymmetry among the studies as demonstrated by the funnel plot above (Fig. 4). This symmetry of studies is considered an indicator of the validity of the results from the studies. One major concern was the allowance for potential confounding factors that may have influenced the incidence of colorectal cancer cases. These factors include physical activity, diet, socioeconomic indicators and among other correlates of colorectal carcinogenesis in women [42]. The fact that using multivariate RRs yielded close to identical pooled estimates to untreated ones shows that the confounding effect of major considered correlates is not likely to be consequential. A significant portion of the data were collected between the year 2000 and 2010 from women with a median age of 58 years and, therefore this information largely refers to oral contraceptive use between the mid-1970s and the mid-1980s. There was no information given on the type of oral contraceptive used back then, but no systematic trend by calendar year was noticed.

Oral contraceptives influence cancer risk through different mechanisms, increasing the risk of some types of cancer e.g breast cancer, while reducing the risk of developing other types e.g colorectal cancer. It has been found that endogenous and exogenous female sex hormones i.e. estrogen and progesterone may lower the risk of colorectal cancers through multiple ways that include: reducing the number and frequency of ovulations females experience throughout her lifetime, therefore, reducing the time of exposure to potentially carcinogenic sex hormones as in the case of ovarian cancer. Second, oral contraceptives may suppress endometrial cell proliferation as in endometrial cancer. Finally, birth control pills reduce the levels of bile acids in the blood, as seen in women under regular oral conjugated estrogens regimens [35]. In this study, reproductive and menstrual factors were evaluated as surrogates for long-term exposure to exogenous and endogenous hormones in several prospective studies targeting colorectal cancer. The incidence of colorectal cancer among women was investigated in relation to reproductive and menstrual factors. This cohort study featured 93,676 women among which 1149 cases of colorectal cancer were reported over a period of 11.9 years. In this study, having had two or more children was inversely correlated with the risk of developing colorectal cancer. Women who had previously used oral contraceptives had a lower risk of colorectal cancer (HR = 0.74, 95% CI: 0.63–0.86); there was, however, no relationship observed between duration of oral contraceptive use and the risk of colorectal carcinogenesis (4 years vs 1 year: HR = 0.94, 95% CI: 0.67–1.32). From the results of the study, it was concluded that prior use of oral contraceptives and parity were the two significant factors that could be directly linked to lower risk of developing colorectal cancer among women of different age groups.

Female hormones confer protection against colorectal carcinogenesis through different mechanisms but chief among them is by influencing changes in bile synthesis and secretion. The overall effect of these changes is a reduced concentration of bile acids in the colon [2]. Other biological mechanisms involved have not been clearly established yet. Estrogens inhibit the in vitro growth of colon cancer cells [43]. Also, it has been shown that estrogen receptors are present in normal and neoplastic mucosal cells of the colon [44]. The estrogen receptor (ER) gene has been shown to play a key role in tumor suppression. This may be the case because the hypermethylation of the promoter region of the ER gene causes deregulated growth in the mucosa of the colon as a result of reduced expression [45]. Estrogenic compounds reduce the concentration of serum insulin-like growth factor-l (IGF-1), a mitogen associated with the increased risk of colorectal cancer [[46], [47], [48]]. Because of all these findings, it is highly likely that the risk of colorectal cancer is inversely related to use of oral contraceptives. Results like these have also been shown by other descriptive epidemiological studies of colorectal cancer [3,4,[4], [4], [5], [6],^39^,^49^], with broad findings of an inverse correlation between colorectal cancer risk and HRT [8,50]. Experimental findings on molecular and physiologic pathways of colorectal carcinogenesis, together with biological hypotheses also support this argument [2]. These findings form the base of informed choices on contraceptives because of better understanding of this potential relation [51]. There are some aspects of oral contraceptive use and risk of colorectal carcinogenesis that remain undefined and need to be further investigated. These include the risk profile with respect to recency and/or duration of use or oral contraceptives, plus possibility of confounding factors. It is, therefore, evident that the problem of causal inference for the observed association still needs to be discussed.

Some of the major strengths of this meta-analysis include the comprehensive analysis of menstrual and reproductive patterns in a range of well-characterised prospective cohort and case control studies featuring more than 2191 verified colorectal cancer cases in 271,222 study participants. The cohort studies also had relatively long follow-up periods of the study participants. Furthermore, the large sample sizes enabled the researchers to carry out stratified analyses with sufficient statistical power, allowing the assessment of women according to waist circumference strata or hormone therapy use in some studies [25], which are some of the factors that may modify the association between colorectal cancer and reproductive history.

A potential drawback of this meta-analysis is that all the primary variables of interest were based on self-reported reproductive history since most of the data was collected mainly through questionnaires and, therefore, the possibility of recall bias cannot be undermined. However, some validation studies have found that self-reported reproductive history shows good agreement with medical records [52,53]. Also, no information on oral contraceptive formulations the women used was available. This information would have been useful to understand the mechanisms responsible for the drop in colorectal cancer incidents. Lastly, there is a possibility that the observed results may have been influenced by survivor bias. This is particularly the case in early life exposures to oral contraceptives like age at menarche. However, no effect modification by factors such as age was observed, and this suggests that the findings were similar in both younger and older women.

## 5 Conclusion

In conclusion, oral contraceptive use is associated with a reduced risk of colorectal cancer. This observation has been backed up by the studies in the meta-analysis which show a reduction in risk ratios for women who had taken oral contraceptives at any point of their lives. There is a need for further research into the biological mechanisms underlying these relationships, however. This will help to better understand potential preventive interventions against colorectal cancer in women. The meta-analysis includes detailed studies, data and information that can be used to inform the strategic use of oral contraceptives in women to reduce the morbidity of cancers in the global population. For instance, the study's major strength is that it provides a comprehensive analysis of menstrual and reproductive patterns in a range of well-characterised prospective cohort and case control studies providing a large data set of more than 2191 verified colorectal cancer cases in 271,222 study participants. Also, the use of both cohort and case-control studies for this meta-analysis provides a broad view of the topic in diverse data sets.

## Ethical approval

Not Applicable (N/A).

## Sources of funding

This research received no specific grant from any funding agency in the public, commercial, or not-for-profit sectors.

## Author contribution

1*.Fadi Abusal: Conceived and designed the analysis and Wrote the paper.

1*.Mohannad Aladwan**:** Conceived and designed the analysis and Wrote the paper.

*: Both authors contributed equally to this paper.

**2. Yazan Al Omari:** Collected the data and Contributed data or analysis tools.

**3. Saja Obeidat:** Collected the data and Contributed data or analysis tools.

**4. Salah Abu warde:** Collected the data and Performed the analysis.

**5. Haya** AlDahdouh**:** Collected the data and Performed the analysis.

**6. Qotadah al shami:** Collected the data.

**7. Qusai:** Collected the data and Performed the analysis.

## Consent

Not Applicable (N/A).

## Registration of research studies

1. Name of the registry: **Not Applicable (N/A**).

2. Unique Identifying number or registration ID: **Not Applicable (N/A**).

3. Hyperlink to your specific registration (must be publicly accessible and will be checked): **Not Applicable (N/A**).

## Guarantor

Fadi Abusal, General Surgery, Al Bashir Hospital, Amman (11151), Jordan. Telephone Number: +962790850815, Email:Dr_fadi@outlook.com.

## Declaration of competing interest

The Authors declare that there is no conflict of interest.
